# Markers of renal fibrosis: How do they correlate with podocyte damage in glomerular diseases?

**DOI:** 10.1371/journal.pone.0217585

**Published:** 2019-06-20

**Authors:** Tiago Giulianni Lopes, Maysa Lucena de Souza, Vinicius Duval da Silva, Mariane dos Santos, William Israel Cardoso da Silva, Thiago Pereira Itaquy, Henrique Iahnke Garbin, Francisco Veríssimo Veronese

**Affiliations:** 1 Postgraduate Program in Medicine: Medical Sciences, Universidade Federal do Rio Grande do Sul, Porto Alegre, Rio Grande do Sul, Brazil; 2 Laboratory of Molecular Biology Applied to Nephrology, Center for Experimental Research, Hospital de Clínicas de Porto Alegre, Porto Alegre, Rio Grande do Sul, Brazil; 3 Barretos Cancer Hospital, Barretos, São Paulo, Brazil; 4 Division of Nephrology, Hospital de Clínicas de Porto Alegre, Porto Alegre, Rio Grande do Sul, Brazil; Peking University First Hospital, CHINA

## Abstract

**Background:**

Renal fibrosis is the result of the interaction of cellular and molecular pathways, which is induced by sustained glomerular injury and involves the podocytes and multiple profibrotic factors. In this study, we investigated the correlation of the mRNA expression of podocyte proteins and profibrotic factors with renal fibrosis measured in renal biopsies of patients with primary and secondary glomerulopathies.

**Methods:**

Eighty-four adult patients with primary or secondary glomerular diseases and 12 controls were included. Demographic and clinical data were collected. Seventy-two percent of the renal biopsies were done less than one year from clinical disease manifestation. The quantification of the podocyte-associated mRNAs of alpha-actinin-4, podocin, and podocalyxin, as well as of the profibrotic factors TGF-β1, CTGF, and VEGF-A were quantified by real-time polymerase chain reaction. The percent positive area of renal fibrosis was measured by immunohistochemistry staining, using anti-CTGF and anti-HHF35 antibodies and unpolarized Sirius Red. Correlations between the expression of tissue mRNAs and the positive area of fibrosis for the measured markers were made by Spearman’s rank correlation coefficient.

**Results:**

In relation to control biopsies, podocyte-specific proteins were downregulated in podocytopathies, in proliferative nephritis, in diabetic kidney disease (DRD), and in IgA nephropathy (IgAN). Messenger RNA of TGF-β1, CTGF, and VEGF-A was upregulated in patients with podocytopathies and in DRD but not in proliferative nephritis and IgAN. Tissue mRNA expression of TGF-β1, CTGF, and VEGF-A were strongly correlated with renal fibrosis, as measured by HHF35; however, the correlation, albeit significant, was moderate for Sirius Red and weak for CTGF. The percent positive area of renal fibrosis measured by Sirius Red was similar between podocytopathies and DRD and significantly higher in podocytopathies compared to IgAN or proliferative nephritis.

**Conclusions:**

In patients with glomerular diseases, the mRNA of TGF-β1, CTGF, and VEGF-A correlated positively with the extent of renal fibrosis, and the positive area of fibrosis was larger in the podocytopathies and in DRD as measured by Sirius Red. The pathways connecting podocyte damage and activation of profibrotic factors to kidney tissue fibrosis need to be better investigated.

## Introduction

Chronic glomerular diseases result in the accumulation of extracellular matrix in the interstitium, referred to as renal tissue fibrosis, that correlates with the loss of kidney function and progressive renal failure [1,2]. Renal fibrosis and global glomerulosclerosis are the histological expressions of chronic damage related to various etiopathogenic mechanisms. It is not entirely clear how these mechanisms develop to induce nephron damage in proteinuric glomerulopathies, but circulating auto-antibodies, pro-inflammatory cytokines and immuno-complex deposition in the initial phase [3], as well as later cellular events and molecular mediators, such as fibrogenic growth factors, pericyte-to-myofibroblast transdifferentiation and apoptosis, are certainly involved [4,5]. Metabolic disorders, oxidative stress, hemodynamic intra-glomerular factors resulting from stimulation of vasoactive molecules, and renal tissue hypoxia are other pathways that also contribute to glomerular and tubulointerstitial fibrosis and the progression of kidney impairment [6].

Podocytes are highly specialized and terminally differentiated epithelial cells that are required for the maintenance of the glomerular filtration barrier and for protein retention. Podocyte foot processes compound an intricate actin cytoskeleton, which is linked to cell-matrix junctions at their basal membrane and to the protein complex forming the slit diaphragm [7]. Podocytes are the main target of injury in primary and in some secondary glomerular diseases, disrupting and detaching from the glomerular basement membrane (GBM) with substantial rearrangement of the actin cytoskeleton. Because podocytes are unable to divide, a gradual podocyte depletion from GBM occurs through the shedding of viable cells into the urinary space. It is still debated if podocytes go on cell death by apoptosis. Kriz et al [8] using transmission electron microscopy were unable to identify apoptotic podocyte cells in the urine; no cases with a nuclear remnant of fragmented or condensed chromatin were found to suggest an apoptotic cell death. Other authors postulate that apoptosis may occur as a consequence of podocytes having lost a connection to a matrix substrate during their passage through the nephron [9], but this is controversial. However, studies have presented convincing evidence that podocyte injury followed by detachment from GBM to Bowman’s space underlies podocytopenia in primary and secondary glomerulopathies, being a marker of glomerular disease activity and progression [8–11].

After initial podocyte damage, there occurs an increase in the mesangial extracellular matrix, GBM thickening, podocyte depletion, and upregulation of profibrotic factors, such as transforming growth factor-beta (TGF-β), inducing epithelial to mesenchymal transition (EMT), fibroblast activation, and detachment and migration of tubular cells to the interstitium [4,12,13]. Connective tissue growth factor (CTGF) is expressed in glomerular podocytes and acts as a mediator of TGF-β actions on mesenchymal cells, and both are co-expressed in several types of glomerular injury, as demonstrated by Ito et al. [14]. Mediators of renal fibrosis act in conjunction by several mechanisms, including cellular events (monocyte and T cell infiltration), by the action of key signalling molecules (NF-κB, TGF-β/Smad), cell apoptosis, and the overproduction of matrix-degrading enzymes [4].

There is still controversy on how to determine the composition of the matrix in fibrotic parenchyma and how to choose the best histological stains to accurately measure the extent of renal fibrosis. In this research, it will be relevant to quantify the expression of glomerular and tubulointerstitial markers of fibrosis, such as CTGF staining for glomeruli, tubules, and interstitial fibroblasts [15], Sirius Red staining for collagen fibres I and III [16–18], and HHF35, an anti-smooth muscle actin antibody, that recognizes myofibroblasts in hyperplastic fibrotic responses [19].

The investigation of the connection between the injured podocyte proteins and the expression of fibrosis markers in kidney biopsies, done in early and more advanced phases of proteinuric glomerulopathies, could add relevant information on how these mechanisms interact. In this study, we correlated the renal tissue mRNA expression of podocyte-specific proteins (alpha-actinin-4, podocin, podocalyxin), growth factors (TGF-β, CTGF), and angiogenic factors (vascular endothelial growth factor, VEGF-A) with the percent positive area of the fibrosis markers CTGF, HHF35, and Sirius Red in kidney biopsies of patients with primary and secondary proteinuric glomerular diseases. Our results showed that the mRNA expression of TGF-β1, CTGF, and VEGF-A correlated positively with the extent of renal tissue fibrosis.

## Materials and methods

### Patients

We included 84 adult patients with primary or secondary glomerulopathies (GP) who were submitted to a kidney biopsy based on clinical indication at the Division of Nephrology of the Hospital de Clínicas of Porto Alegre (HCPA), from March 2012 to December 2016. The biopsies were classified according to histological diagnosis and divided in four groups as follows: 1) Podocytopathies (n = 44): focal and segmental glomerulosclerosis, FSGS (n = 24), membranous nephropathy, MN (n = 13), minimal change disease, MCD (n = 7); 2) Proliferative nephritis (n = 15): lupus nephritis, LN (class III: n = 4, class IV: n = 6), ANCA-associated crescentic glomerulonephritis, CrescGN (n = 5); 3) Diabetic kidney disease, DRD (n = 13); and 4) IgA nephropathy, IgAN (n = 12). In patients with DRD, the Tervaert pathological classification [20] was defined as follows: class I: n = 2; class IIa: n = 2; class IIb: n = 3; class III: n = 5; and class IV: n = 1.

For the control group, 12 patients who underwent nephrectomy for a localized renal tumour were included, and we used the non-affected part of the renal tissue. These biopsy samples did not present evidence for any other renal disease. Controls were defined as healthy when they reported no personal or familial history of kidney disease, blood pressure levels were less than 140/90 mmHg, eGFR was higher than 90 mL/min/1.73 m^2^, there was no active urinary sediment or proteinuria on urinalysis, and the measured proteinuria was less than 200 mg/24 hours.

This study was approved by the HCPA Research Ethics Committee and registered with the Institutional Review Board under number 00000921. All study procedures were performed in compliance with the 1975 Declaration of Helsinki, and all patients and controls provided written informed consent for participation.

### Clinical data

The following demographic and clinical data were collected from the electronic medical records: age, sex, ethnicity, systolic and diastolic blood pressure, time between onset of clinical manifestations of GP and renal biopsy (in months), serum creatinine, estimated glomerular filtration rate (eGFR) by the CKD-EPI equation, serum albumin, total cholesterol, HDL and LDL cholesterol, triglycerides, and proteinuria measured in g/24 hours or in a random sample (urinary protein/creatinine ratio, mg/dL/mg/dL). The presence of edema, non-nephrotic proteinuria, nephrotic syndrome, hematuria and cellular casts in the qualitative examination of urine, nephritic syndrome, and acute renal injury or chronic kidney disease (CKD) attributable to glomerular disease were considered as clinical manifestations of GP.

### Renal biopsies

Renal biopsy was performed by ultrasound guidance, with an automated needle Pro Magnum (Bard, Covington, GA, USA) with a 16-G needle and processed for light microscopy and immunofluorescence in the Pathology Division. Two fragments were obtained, and one-fourth of the second fragment was frozen at -80° C for molecular analyses. The slides were stained with hematoxylin and eosin, periodic acid-Schiff, silver methenamine and Masson trichrome. The histopathological diagnosis was made by a nephropathologist during their routine care, and included assessment of the total number of glomeruli, the total number of globally sclerosed glomeruli, the presence of synechiae, mesangial matrix expansion and hypercellularity, endocapillary hypercellularity, GBM thickening, the presence and number of crescents, and the presence of focal or diffuse inflammatory interstitial infiltrate. The percentage of interstitial fibrosis and tubular atrophy (IFTA) were quantified in the renal cortex with Masson’s trichrome staining, based on the following stratification: absent (<5%), mild (5–25%), moderate (26–50%), or severe fibrosis (>50%). In the renal biopsies of the controls, to be considered a normal tissue, the glomeruli had to have normal cellularity, normal GBM, preserved endothelial and podocyte cells, normal mesangial matrix and cellularity, a normal tubular and interstitial segment with an absence of vascular alterations, and no evidence of tumour in the fragment examined.

### Quantification of podocyte-associated mRNAs in kidney tissue

Podocyte-associated messenger RNAs (mRNAs) of alpha-actinin-4, podocin, and podocalyxin were quantified using real-time polymerase chain reaction (RT-PCR) in renal biopsies, as previously described [21]. The expression of these mRNAs in kidney tissue was defined in relation to the mRNA levels detected in controls. In brief, mRNA was extracted from tissue samples using the QIAamp RNA Blood Mini Kit (Qiagen Inc. Chatsworth, CA, USA), and reverse RNA transcription was performed using the High-Capacity cDNA Kit (Applied Biosystems, Foster City, CA, USA) in accordance with the manufacturer’s instructions. The final volume of 20 μL purified RNA was stored at –20°C. RT-qPCR was performed using a TaqMan Universal PCR Master Mix. Specific primers (Applied Biosystems, Foster City, CA, USA) to the following genes were used: NPHS2, podocin (ID: Hs00387817_m1); alpha-actinin-4 (ID:Hs00245168_m1), podocalyxin (ID:Hs01574644_m1); CTGF (ID: Hs00170014_m1); TGF-β1 (ID: HS00998133_m1); and VEGF-A (ID:HS00173626_m1). Additionally, 18s rRNA (TaqMan PDAR, Foster City, CA, USA) was used as an endogenous control for sample normalization. RT-qPCR was performed in duplicate in 48-well plates containing 2 μL cDNA. Data were collected in a Step One real Time PCR systems (Applied Biosystems, Foster City, CA, USA) using the following cycling parameters: 95°C for 10 minutes, followed by 40 cycles at 95°C for 15 seconds and 60°C for 60 seconds. Relative quantification of target gene expression was performed using the 2^-ΔΔCt^ comparative method, in which threshold cycle numbers (CT) were determined by the point at which a statistically significant increase in fluorescence was detected.

### Immunohistochemistry for markers of renal fibrosis

To prepare the slides, renal tissue sections of 3-μm thickness were obtained with a microtome (Leica SM 2000R, Wetzlar, Germany) and were placed on slides pretreated with Histogrip (Thermo Fischer Scientific, Waltham, Massachusetts, USA), and taken to the oven at 60° C for 24 hours. The slides were dewaxed three times by incubation in xylene for 10 minutes, followed by rehydration in ethanol at decreasing concentrations, starting with absolute ethanol 90%, 80% and 70% for 3 minutes at each dilution. The sections were then washed three times in distilled water.

Antigen exposure through heat-induced antigen recovery was performed by incubating the slides in a Coplin jar with Target Retrieval Solution buffer, pH 9.0 (Dako, Agilent Pathology Solutions, Santa Clara, CA, USA), placed in water bath at 99° C for 40 minutes, and then cooled for 20 minutes at room temperature; the slides were then washed in PBS buffer, pH 7.2. The blockade of endogenous peroxidase was performed with H_2_O_2_ 3% solution in methyl alcohol, in two 15-minute incubations, followed by three wash cycles with PBS buffer. Nonspecific binding was blocked with a serum-free protein-blocking solution (Dako, Agilent Pathology Solutions, Santa Clara, CA, USA) for 30 minutes at room temperature. The sections were incubated by the capillary method through the Thermo Scientific Sequenza Manual Staining system (Thermo Fischer Scientific, Kalamazoo, Michigan, USA) immunostaining station overnight at 2° C to 6° C and diluted in antibody diluent with background reducing components (Dako, Agilent Pathology Solutions, Santa Clara, CA, USA) for the following antibodies: anti-CTGF (Santa Cruz Biotechnology, Inc., Dallas, TX, USA) at 1:50 dilution and anti-HHF35 (Santa Cruz Biotechnology, Inc., Dallas, TX, USA) at 1:100 dilution. After incubation with the primary antibody, the sections were washed three times in PBS buffer. For amplification of the antigen-antibody reaction of HHF35, the Advance HRP system (Dako, Agilent Pathology Solutions, Santa Clara, CA, USA) was used according to the manufacturer’s instructions. For CTGF, the rabbit anti-goat horseradish peroxidase, HRP (Abcam, Cambridge, Massachusetts, USA) was used. The slides were then washed in PBS buffer and incubated with diaminobenzidine solution DAB Substrate Chromogen System (Dako, Agilent Pathology Solutions, Santa Clara, CA, USA) for 5 minutes. After washing in distilled water, the slides were stained with Harris Hematoxylin for 1 minute, washed in running water until complete removal of the dye and incubated in a 37 mM ammonia solution for 15 seconds. Finally, the slides were dehydrated in absolute ethyl alcohol (four incubations of 2 minutes) followed by two treatments with xylene for 5 minutes. The slides were mounted with synthetic Entellan medium (Merck KGaA, Darmstadt, Germany). CTGF and HHF35 positive areas of fibrosis were stained brown, both in the glomeruli and in the tubulointerstitium.

For detection of type I and type III collagen fibrils, Sirius Red (Merck KGaA, Darmstadt, Germany), at a concentration of 0.1% in a saturated solution of picric acid, was used. Histological sections of 4 μm were deparaffinized and hydrated with running water. Afterward, the sections were incubated for 1 hour in a 0.1% Sirius Red solution and then washed in running water until removal of the dye, dehydrated in alcohol, diaphanized in xylol, and mounted with synthetic mounting medium. The positive areas of fibrosis were stained a strong red colour and were quantified under unpolarized light in the glomeruli and in the tubulointerstitium.

### Quantification of renal tissue fibrosis

Renal tissue fibrosis was quantified on HHF35, CTGF- and Sirius Red-stained slides with an Olympus BX40 microscope (Olympus, Tokyo, Japan), and a Moticam CMOS 3.0MP camera (Motic Asia, Hong Kong, China) connected to an Intel Windows 7 Microsoft PC (Redmond, Washington, USA). Non-overlapping images were obtained from the whole tissue of each slide (magnification 100x) and the quantification was performed with the software Image Pro Plus 7.0.1 (Media Cybernetics, Rockville, USA). For all fibrosis markers, the areas were selected and segmented by the software with a fixed-colour threshold set as previously defined, and the resulting sum was matched to the total tissue area obtained from light microscopy images of each slide.

### Statistical analysis

Data were presented as absolute or relative frequencies, mean and standard deviation (SD), or median and interquartile ranges (IQR). The normality of the variables was established by the Shapiro-Wilk test. Categorical variables were analysed using the Chi-squared test or Fisher’s exact test, as appropriate. Significantly skewed variables were logarithmically transformed to reduce asymmetry. Differences between groups were analysed by independent t tests (symmetric variables) and Mann-Whitney or Kruskal-Wallis tests (asymmetric variables), according to the number of groups. Adjustments for multiple statistical comparisons were made by Dunn’s test with Bonferroni correction. The comparison of differences in expression of log10 mRNA of each podocyte protein among podocytopathies, proliferative nephritis, diabetic kidney disease and IgA nephropathy was presented as median and IQR in boxplots. Spearman’s rank correlation coefficient was used to test the correlation between the renal tissue expression of podocyte mRNAs with the percent positive area of fibrosis for each measured marker and are presented as scatterplots. All analyses were performed by SPSS for Windows software (version 21.0, SPSS Inc., Chicago, IL). The level of significance was set at P less than 0.05.

## Results

### Demographic and clinical data

The demographic and clinical characteristics of the study sample are presented in Table 1. The mean patient age was 45±15 years and the participants were mostly women (58%) and Caucasians (89%). The mean initial eGFR of 53 (29–90) ml/min/1.73 m^2^ but had a large variation (12 to 148 ml/min/1.73 m2). The median between the onset of clinical manifestations and renal biopsy was 4 (2–18) months. Forty-one percent of the patients were biopsied less than 3 months, 31% between 3–12 months, and 28% after 12 months since their initial clinical presentation. The median follow-up duration was 21 months (range 9–42).

**Table 1:** Demographic and clinical characteristics of the patients.

	Patients (N = 84)
Age (years)	45±15
Sex (female)	49 (58)
Race (white)	75 (89)
Time to biopsy (months)	4 (2–18)
eGFR (first / last)	53 (29–90) / 39 (21–76)*
UPCR, mg/mg (first / last)	3.5 (1.5–5.7) / 1.0 (0.3–2.5)#
Serum albumin (g/dL)	3.35 (2.85–3.90)
Total cholesterol (mg/dL)	242±63
LDL cholesterol (mg/dL)	154±52
Glucose (mg/dL)	96±22
Follow-up time (months)	21 (9–42)
**Clinical outcomes**	
Without dialysis	51 (61)
End-stage renal disease/dialysis	20 (24)
Loss of follow-up	7 (8)
Death	6 (7)

mean±SD; n(%); median (IQR); eGFR: estimated glomerular filtration rate (ml/min/1.73 m^2^); UPCR: urine protein/creatinine ratio;

*p = 0.009;

^#^p<0.001

Twenty (20%) patients progressed to end-stage renal disease (ESRD) and initiated dialysis, while 51(61%) remained on conservative treatment with preserved renal function. Comparing patients not on dialysis with those with ESRD, mean baseline proteinuria was 3.8 (range, 1.9–5.5) vs. 4.7 (range, 2.8–6.6), respectively (p = 0.460), and, after immunosuppressive therapy (some on induction but most under maintenance treatment), the mean proteinuria at the last follow-up was 0.5 (range, 0.2–1.5) vs. 3.8 (range, 1.7–9.7), respectively (p<0.001). At the same time, points, the renal function measured by eGFR in patients not on dialysis compared to patients with ESRD was 57 (range, 42–103) vs. 24 (range, 15–35), respectively (p<0.001), and the final eGFR was 64 (range, 31–95) vs. 8 (range, 5–14) mL/min/1.73 m^2^ respectively (p<0.001).

### Renal biopsy findings

Overall, the predominant glomerular changes were mesangial matrix expansion (68%) and mesangial cell hypercellularity (50%), as presented in Table 2. Thickening of the GBM and synechiae were found in 45% and 55% of biopsies, respectively. A focal or diffuse interstitial inflammatory infiltrate was described in 57% of the biopsies. In 42% of the biopsies, more than 30% of the glomeruli were globally sclerosed, in 57% there were mild IFTA (5–25% of the fragment), and moderate (26–50%) or severe (>50%) IFTA was described in 37%.

**Table 2:** Histopathological findings of the renal biopsies.

Microscopic findings	Patients (N = 84)
**Glomerular Changes**	
Total number of glomeruli	14 (10–19)
Globally sclerosed glomeruli (%)	13 (0–30)
*Less than 30%*	49 (58)
*Equal or greater than 30%*	35 (42)
Mesangial matrix expansion	57 (68)
Mesangial hypercellularity	42 (50)
Endocapillary hypercellularity	18 (21)
GBM thickening	38 (45)
Synechia	46 (55)
Crescentic glomerulonephritis	10 (12)
**Tubulointerstitial changes**	
Inflammatory interstitial infiltrate	
*Absent*	36 (43)
*Focal / diffuse*	35 (42) / 13 (15)
Interstitial fibrosis / tubular atrophy (IFTA)	
*Absent*	5 (6)
*Mild*	48 (57)
*Moderate*	20 (24)
*Severe*	11 (13)
**Vascular changes**	
Fibrous intimal thickening	56 (67)
Hypertrophy of smooth muscle cells	20 (24)

median (IQR); n(%); GBM: glomerular basement membrane; IFTA: absence: <5%; mild: <5–25%; moderate: 26–50%; or severe: >50%.

### Expression of mRNA of podocyte-associated proteins and profibrotic factors in glomerular diseases and in normal controls

**Fig 1:**
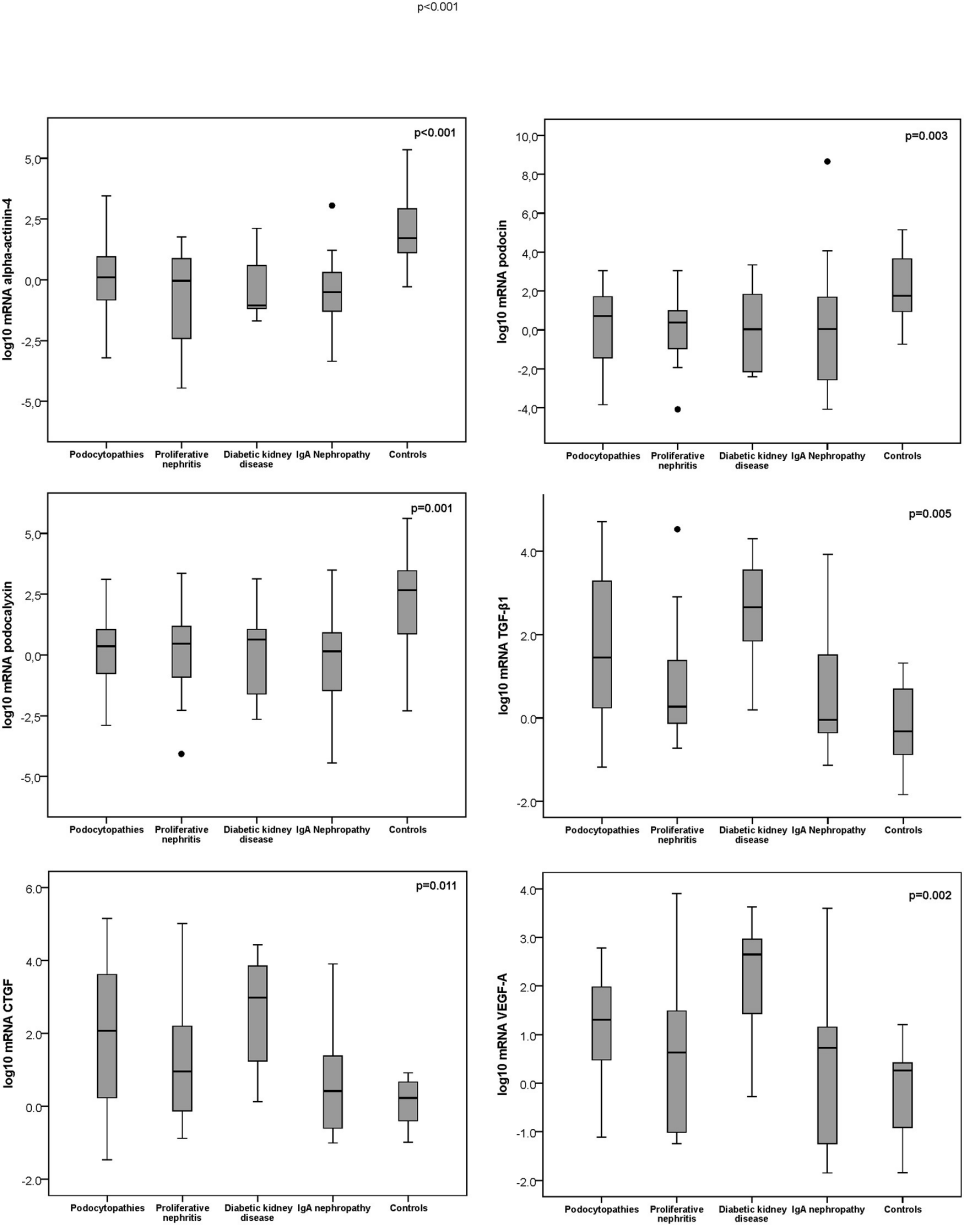
Boxplots of logarithmic transformed medians and 25^th^ to 75^th^ percentiles of the quantification levels of normalized mRNA (2^-ΔΔCt^) of alpha-actinin-4, podocin, podocalyxin, TGF-β1, CTGF, and VEGF-A kidney tissue expression according to each group of glomerular disease relative to the mRNA (2^-ΔΔCt^) expression measured in control biopsies. For each graphic showing the level of podocyte protein or profibrotic factor expression, the p-values as calculated by the Kruskal-Wallis test are presented.

In Fig 1, boxplots represent the distribution of the log-transformed mRNA (2^-ΔΔCt^) of alpha-actinin-4, podocin, podocalyxin, TGF-β1, CTGF, and VEGF-A for each glomerulopathy in relation to the mRNA expression in control biopsies, based on the minimum, 25th percentile, median, 75th percentile, and maximum values; the upper and lower whisker indicate the highest and lowest value (Kruskal-Wallis test). In relation to control biopsies, podocyte-specific proteins were downregulated in every glomerular disease, however the expression of profibrotic factors was upregulated in patients with podocytopathies and diabetic kidney disease, but not in patients with proliferative nephritis or IgA nephropathy.

Relative to controls, patients with podocytopathies showed a downregulation of the expression of log10 mRNA of alpha-actinin-4 (p = 0.001), podocin (p = 0.009), podocalyxin (p = 0.003), and an upregulation of TGF-β1 (p = 0.016), CTGF (P = 0.025), and VEGF-A (p = 0.018), as analysed by the Mann-Whitney test (Fig 1). In patients with DRD, log10 mRNA expression showed a similar behaviour for alpha-actinin-4 (p = 0.004), podocin (p = 0.039), podocalyxin (p = 0.019), and for TGF-β1 (0.016), CTGF (0.007) and VEGF-A (0.025). In proliferative nephritis, the log10 mRNA expression of alpha-actinin-4 (p = 0.002), podocin (p = 0.008), and podocalyxin (p = 0.026) differed from the controls, but not the molecules related to renal fibrosis (TGF-β1, CTGF, and VEGF-A: p>0.05). Similarly, in IgA nephropathy the log10 mRNA expression of alpha-actinin-4 (p = 0.001), podocin (p = 0.012), and podocalyxin (p = 0.02) was significantly higher, but, for the profibrotic factors, there was no significant difference (p>0.05). The mRNA expression of TGF-β1, CTGF, and VEGF-A was significantly higher in DRD when compared to proliferative nephritis (p = 0.029, p = 0.032, and p = 0.04, respectively) and to IgAN (p = 0.017, p = 0.037, and p = 0.025, respectively), but not for the podocytopathies (p>0.05 for all comparisons).

### Histologic assessment of renal fibrosis by specific markers as measured by immunohistochemistry staining

**Fig 2:**
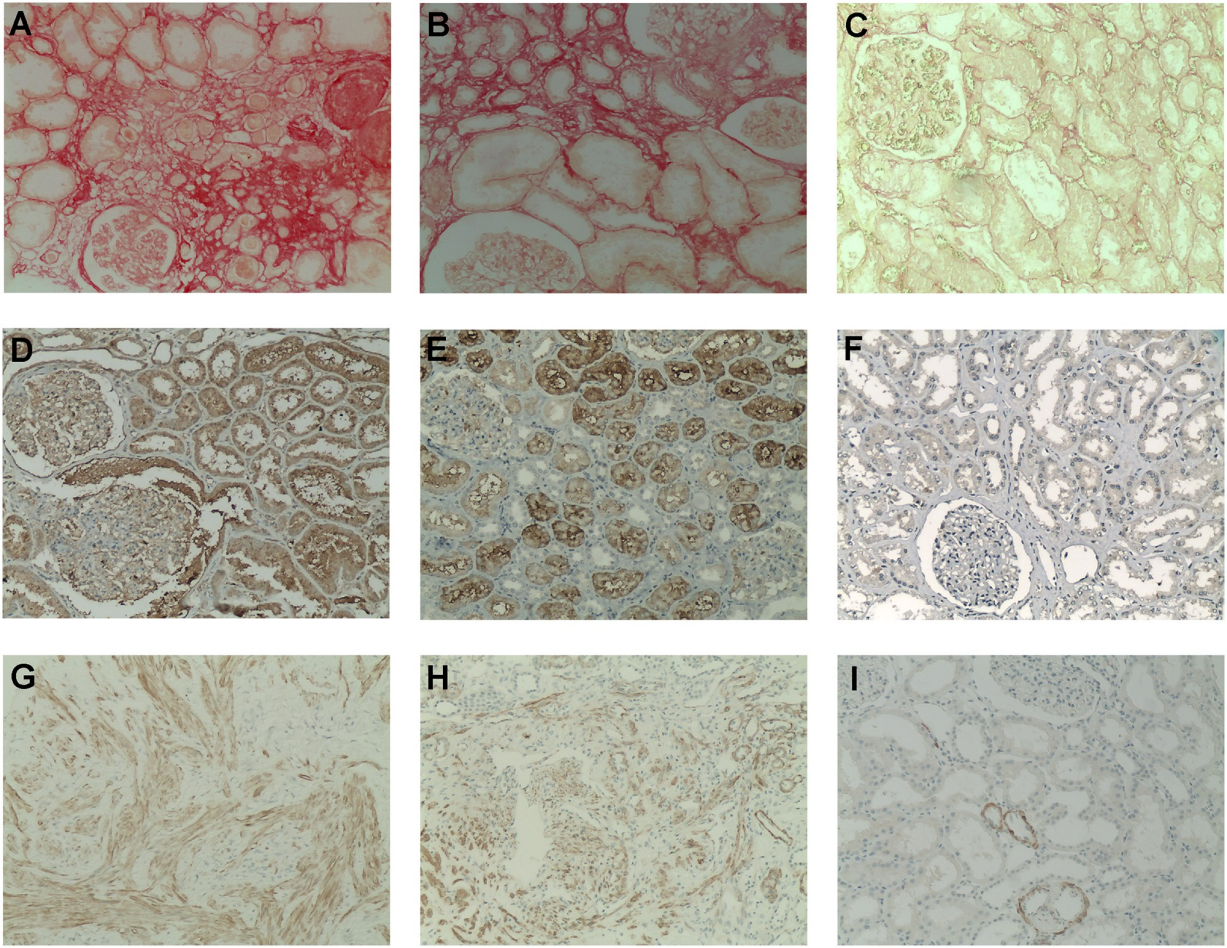
Immunohistochemistry in kidney biopsies for the histologic assessment of the renal fibrosis by Sirius Red, CTGF, and HHF35 staining. Sirius Red (unpolarized; all images with magnification x100): (A) Strong red staining of collagen fibers in the interstitium and in the glomeruli. (B) Moderate staining of collagen fibers distributed in the interstitium and a weak staining in the glomeruli. (C) No staining was detected in a control biopsy. CTGF (diaminobenzidine; all images with magnification x100): (D) Moderate to intense staining expressed in podocytes and along the glomerular capillary walls, very strong staining expressed in tubules, and a very weak staining in the interstitium. (E) Moderate staining in the tubules, weak in the glomeruli, and no staining in the interstitium. (F) No staining in the glomeruli and in the interstitium, and a very weak staining in a few tubules detected in a control biopsy. HHF35 (diaminobenzidine, all images with magnification x100): (G) Strong staining of smooth muscle bundles with cellular intimal thickening and intimal fibrosis in the cortical interstitium, and to a lesser degree, in the tubules and in the glomerular capillary walls. (H) Moderate staining in the tubules, interstitium, and in a glomerulus. (I) Absence of or a scarce staining in a few tubules in a control biopsy.

In Fig 2, representative renal tissue expression of the fibrosis markers Sirius Red, CTGF and HHF35 are presented. As a collagen-specific marker, Sirius Red stained collagen fibrils in the interstitium, in areas of intimal fibrosis, and in the glomeruli and arteries. In Fig 2A, there is an intense red staining in the glomeruli and in the interstitium, and in Fig 2B, a moderate staining was distributed in the interstitium with a weak staining in the glomeruli in kidney biopsies diagnosed as FSGS (Fig 2A) and DRD (Fig 2B), respectively. In Fig 2C, there is an absence of staining in a control biopsy.

CTGF was moderately to intensely expressed in podocytes and along the glomerular capillary walls, and it was very strongly expressed in tubules—but less in the interstitium—in DRD (Fig 2D) and IgA nephropathy (Fig 2E). Absent or very weak CTGF staining was found in control biopsies (Fig 2F). In podocytopathies, specifically in focal segmental glomerulosclerosis and in membranous nephropathy, CTGF was also strongly expressed in the glomeruli and in the tubulointerstitial areas (data not shown).

HHF35, a marker of smooth muscle actin and of arteriolar intimal fibrosis, was strongly expressed in the cortical interstitium and, to a lesser degree, in the tubules and in the glomerular capillary walls (Fig 2G and 2H), reflecting the positive smooth muscle bundles with cellular intimal thickening and intimal fibrosis. The images in Fig 2G and 2H represent the renal biopsy of patients with MN and FSGS, respectively. As shown in Fig 2I, absent or scarce HHF35 staining was detected in control kidney biopsies.

### Correlation between the expression of podocyte proteins and profibrotic factors with the percent positive area for fibrosis in the kidney tissue

In Table 3 the median and interquartile ranges of the percent positive area of fibrosis of the three markers for each group of glomerular disease, relative to the percentage of fibrosis measured in control biopsies, are shown with the respective p-value according to Mann-Whitney calculations. Sirius Red had the strongest and most extensive positive area of fibrosis, followed by CTGF and HHF35, in sequence.

**Table 3:** Median and interquartile ranges of the percent positive area of fibrosis in renal biopsies by staining in each glomerular disease in relation to control biopsies and between pairs of glomerular diseases.

Glomerular disease	Percent positive area of fibrosis					
	Sirius Red	p-value^1^	CTGF	p-value	HHF35	p-value
Podocytopathies	15.3 (6.2–39.4)^2^	<0.001	7.1 (3.6–10.5)	<0.001	1.4 (0.4–3.8)	0.018
Proliferative nephritis	5.1 (1.7–13.5)	0.001	3.9 (1.2–7.7)	0.003	1.0 (0.3–3.1)	0.043
Diabetic kidney disease	13.9 (5.4–34.7)	<0.001	6.5 (2.9–10.2)	<0.001	0.6 (0.2–2.6)	0.205
IgA nephropathy	9.0 (3.5–20.6)	0.001	4.5 (2.3–8.7)	0.001	0.4 (0.06–2.1)	0.162
Control biopsies	0.13 (0.07–0.44)	-	0.03 (0.01–0.19)	-	0.14 (0.03–0.23)	-
Interstitial inflammatory infiltrate		0.190		0.049		0.484
*Absent*	10.9 (5.1–35.9)		7.2 (4.0–9.6)		0.8 (0.2–2.5)	
*Focal / diffuse*	10.4 (2.3–19.2)		4.6 (1.3–8.7)		0.9 (0.3–3.4)	

^1^Glomerular diseases relative to controls;

^2^Median (IQR); Comparison between two groups of glomerular disease (by pairs): Sirius Red staining: podocytopathies vs. diabetic kidney disease (p = 0.169); podocytopathies vs. proliferative nephritis (p = 0.028), and podocytopathies vs. IgA nephropathy (p = 0.037).

There was no difference for CTGF and HHF35 staining comparing podocytopathies vs. diabetic kidney disease (p = 0.185 and 0.318, respectively), podocytopathies vs. proliferative nephritis (p = 0.440 and 0.072, respectively), and vs. IgA nephropathy (0.451 and 0.348, respectively). IFTA: interstitial fibrosis and tubular atrophy (absent: <5%; mild: <5–25%; moderate: 26–50%; or severe: >50%) vs. interstitial inflammatory infiltrate: absent: 10 (5–25) vs. focal /diffuse: 15 (10–45); p = 0.073.

Relative to control biopsies, the percent positive area of renal fibrosis measured by Sirius Red was higher in podocytopathies and in DRD, intermediate in IgAN, and lower in proliferative nephritis. The same behaviour was seen for the CTGF staining. HHF35 staining was significant only for podocytopathies and for proliferative nephritis. Comparing the glomerular diseases by pairs and for each marker of fibrosis, we found a statistically significant difference for Sirius Red staining between podocytopathies vs. proliferative nephritis (p = 0.028) and podocytopathies vs. IgAN (p = 0.037), as shown in Table 3. There was no difference when comparing the podocytopathies vs. DRD (p = 0.169) for Sirius Red (p = 0.169), for CTGF (p = 0.185) and also for HHF35 (p = 0.318).

**Fig 3:**
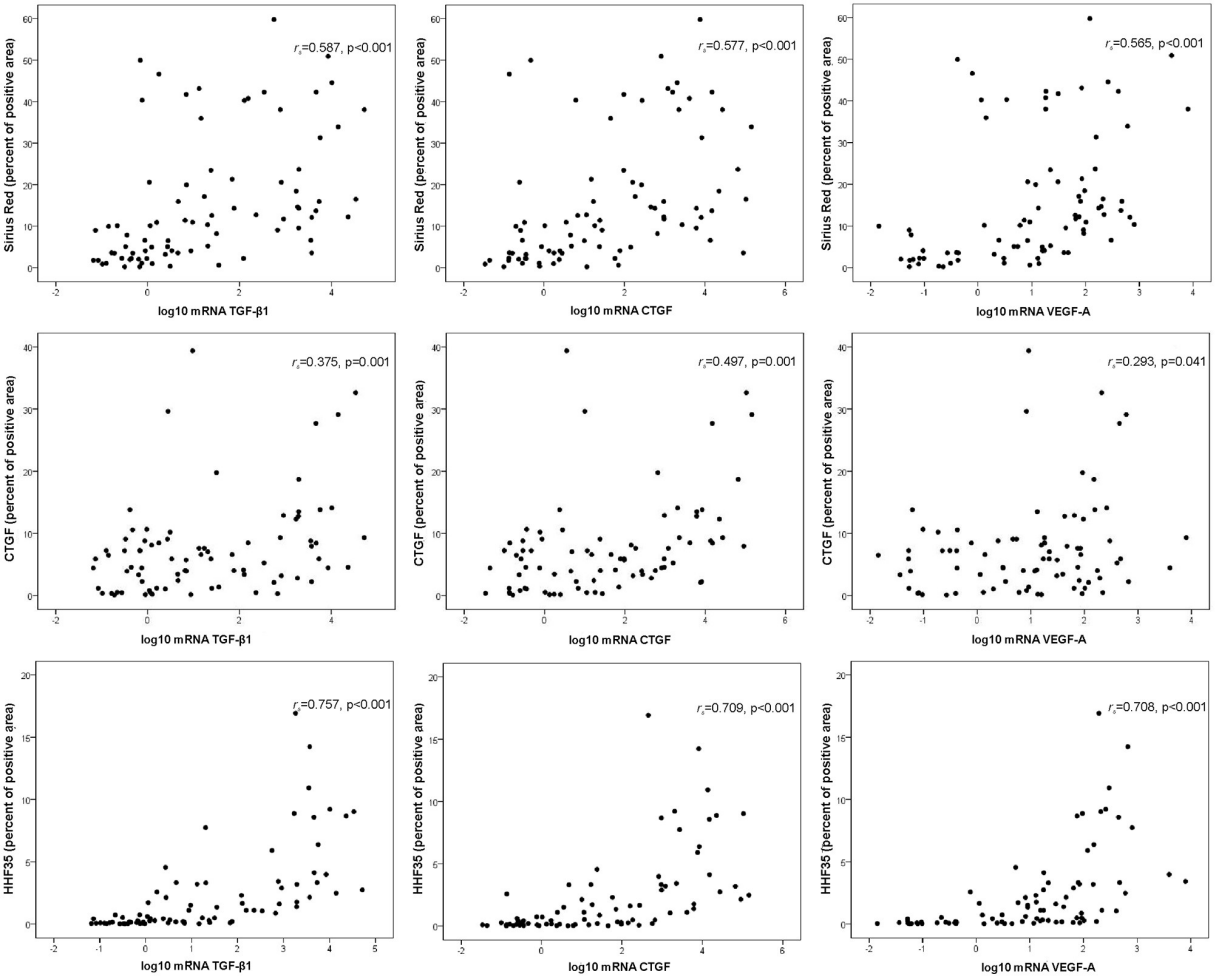
Spearman’s correlations between the percent positive area of fibrosis measured by HHF35, CTGF, and Sirius Red staining with the log transformed mRNA (2^-ΔΔCt^) of TGF-β1, CTGF and VEGF-A, as expressed in renal biopsies of patients diagnosed with podocytopathies, proliferative nephritis, diabetic kidney disease, and IgA nephropathy. In each graphic, the correlation coefficient *r*_*s*_ and respective p-values are presented. The three rows all have different y-axis scales.

To identify statistically significant relationships between the variables, Spearman correlations were calculated between the positive areas of fibrosis by Sirius Red, CTGF, and HHF35 in correlation with the log10 mRNA of TGF-β1, CTGF and VEGF-A expressed in kidney biopsies (Fig 3). The log10 mRNA of TGF-β1, CTGF and VEGF-A were positively and significantly correlated with Sirius Red, CTGF and HHF35. HHF35 showed the strongest correlations with the mRNA expression of TGF-β1, CTGF and VEGF-A, as presented in Fig 3. However, the correlation, although statistically significant, was moderate for Sirius Red and weak for CTGF. The positive area of fibrosis as measured by HHF35 significantly correlated with the expression of log10 mRNA of TGF-β1, VEGF-A, and CTGF respectively, for podocytopathies (*r*_*s*_ = 0.706, p<0.001; *r*_*s*_ = 0.823, p<0.001; and *r*_*s*_ = 0.650, p<0.001), proliferative nephritis (*r*_*s*_ = 0.719, p = 0.004; *r*_*s*_ = 0.868, p<0.001; and *r*_*s*_ = 0.767, p = 0.001), and IgA nephropathy (*r*_*s*_ = 0.841, p = 0.001; *r*_*s*_ = 0.802, p = 0.002; and *r*_*s*_ = 0.666, p = 0.018). For diabetic kidney disease, there was a significant correlation for TGF-β1 (*r*_*s*_ = 0.863, p<0.001), but only a trend for VEGF-A (*r*_*s*_ = 0.489, p = 0.086) and for CTGF (*r*_*s*_ = 0.516, p = 0.071).

As expected, the percentage of interstitial fibrosis and tubular atrophy (IFTA), as quantified in the renal cortex by the nephropathologist (data not shown), did not correlate with TGF-β1 (r = 0.129; p = 0.250) and CTGF (r = 0.156; p = 0.163), but showed a weak correlation with VEGF-A (r = 0.221; p = 0.048).

**Fig 4:**
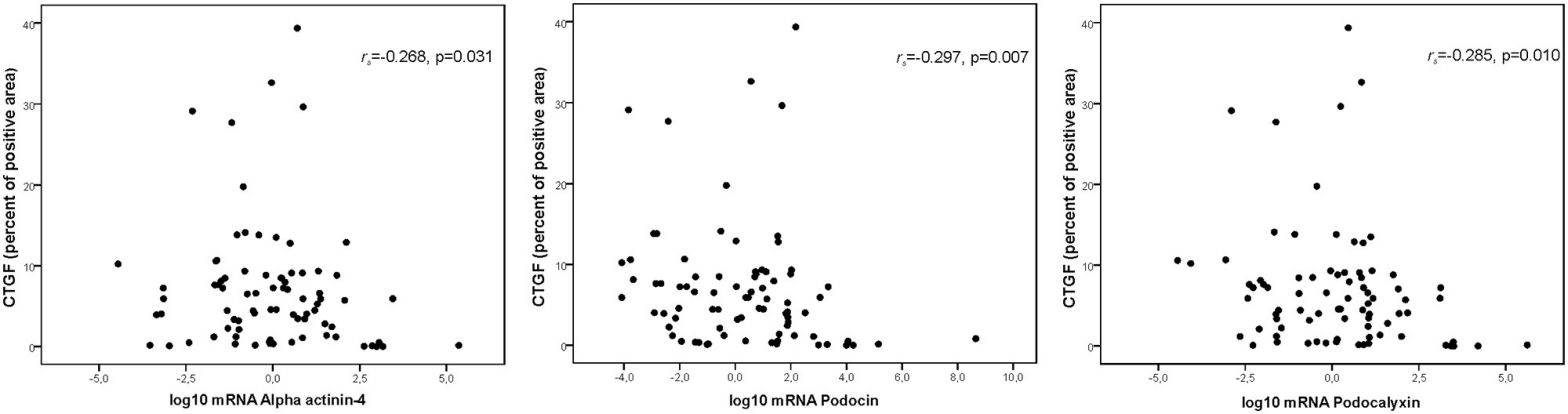
Spearman’s correlations between the percent positive area of fibrosis measured by CTGF staining and the log transformed mRNA (2^-ΔΔCt^) of alpha-actinin-4, podocin, and podocalyxin, as expressed in renal biopsies of patients diagnosed with podocytopathies, proliferative nephritis, diabetic kidney disease, and IgA nephropathy. In each graphic, the correlation coefficient *r*_*s*_ and respective p-values are presented.

An inverse correlation between the percent positive area of interstitial fibrosis and the log10 mRNA of alpha-actinin-4, podocin, and podocalyxin was found, but the only statistically significant correlation was observed for CTFG (and not for Sirius Red or HHF35), as shown in Fig 4. For HHF35, the correlations were: alpha-actinin-4 *r*_*s*_ = -0.155, p = 0.165; podocin: *r*_*s*_ = -0.199, p = 0.074, and podocalyxin: *r*_*s*_ = -0.127, p = 0.255. For Sirius Red, alpha-actinin-4 *r*_*s*_ = -0.018, p = 0.873; podocin: *r*_*s*_ = -0.069, p = 0.537, and podocalyxin: *r*_*s*_ = -0.134, p = 0.230.

### Association between the interstitial cellular infiltrate with the positive area for fibrosis in renal biopsies

The inflammatory cellular infiltrate in the renal interstitium was categorized as absent or focal/diffuse, as there were no differences when analysing the infiltrate separately as focal or diffuse. These data are presented in Table 3. We found a weak association for CTGF (p = 0.049), but not for Sirius Red (p = 0.190) or for HHF35 (p = 0.484). A trend for association between the interstitial cellular infiltrate with the percentage of IFTA was also found (p = 0.073).

### Correlation between the expression of podocyte proteins, profibrotic factors and fibrosis markers with kidney function

**Fig 5:**
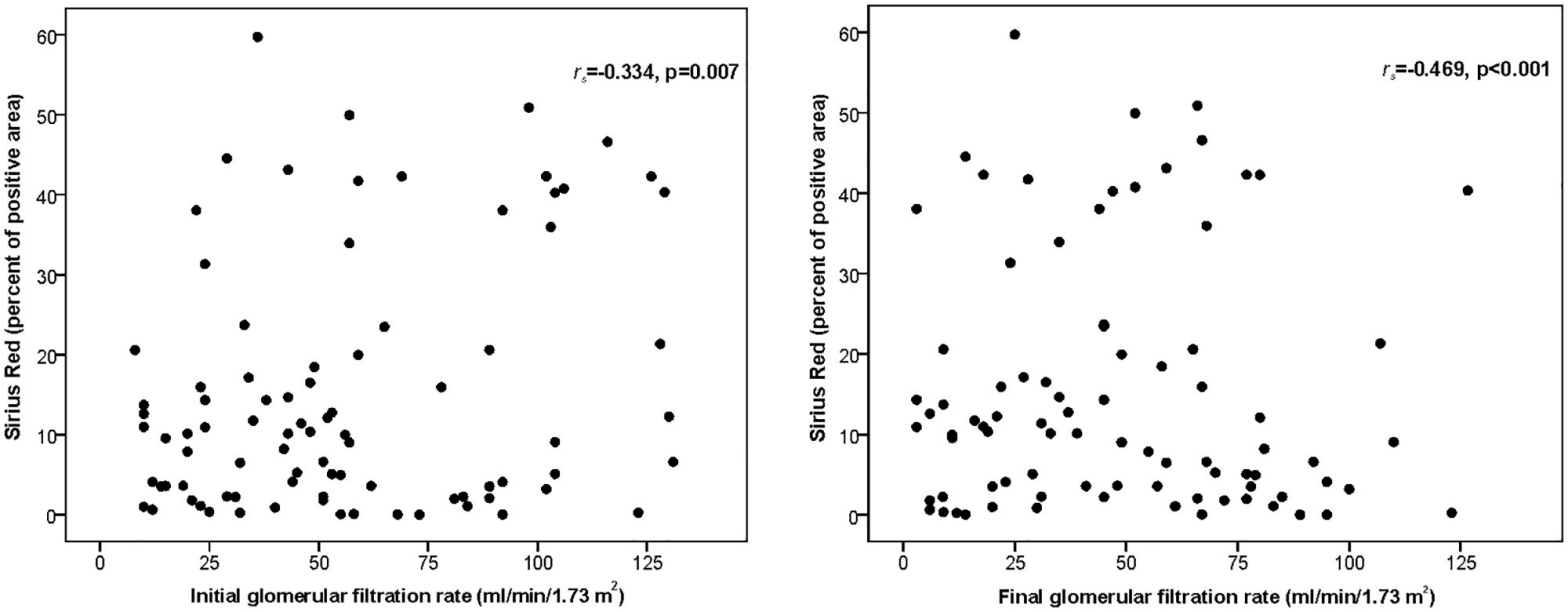
Spearman’s correlations between the percentage positive area of fibrosis measured by Sirius Red staining with renal function, as estimated by glomerular filtration rate at the time of renal biopsy (initial) and at last follow up (final). In each graphic, the correlation coefficient *r*_*s*_ and respective p-values are presented.

Spearman´s correlations were done between podocyte-associated proteins and pro-fibrotic factors with renal function, as measured by eGFR at the time of renal biopsy (initial) and at last follow up (final). No significant correlation was found for alpha-actinin-4, podocin, podocalyxin, TGF-β1, CTGF, and VEGF-A. Likewise, the positive area of fibrosis as quantified by Sirius Red, CTGF and HHF35 was correlated with the initial and final eGFR. There was found statistical significance but only for Sirius Red, with a moderate negative correlation with both initial eGFR (rs = -0.334, p = 0.007) and final eGFR (rs = -0.469, p<0.001), as shown in Fig 5.

## Discussion

In this study, we analysed the correlation between the mRNA expression of podocyte proteins, profibrotic and angiogenic factors and the percentage of renal tissue fibrosis, as quantified by three different stains in kidney biopsies of patients with glomerular diseases. In relation to the expression of TGF-β1, CTGF and VEGF-A, and the extent of renal fibrosis, we found a positive and significant correlation that was strong for HHF35, moderate for Sirius Red, and weak for CTGF. In addition to that, an inverse correlation, which was significant for CTGF, was observed between alpha-actinin-4, podocin, and podocalyxin mRNA expression and the percentage of renal fibrosis. Renal fibrosis, as measured by Sirius Red, was more extensive in podocytopathies and DRD, probably reflecting the progressive podocyte injury and podocytopenia of glomerular diseases in whom podocytes are most affected, which triggers several mechanisms of tissue fibrosis in the glomeruli, tubulointerstitium, and the vasculature.

The pathologic findings of renal fibrosis are described as glomerulosclerosis, tubule-interstitial fibrosis, and loss of renal parenchyma where tubular atrophy, capillary loss, and podocyte depletion are prominent [4]. As described by Kaissling et al [22], the progression of the disease in the tubule is secondary to that in glomerulus, as the injury of a glomerular segment progress to Bowman’s capsule forming synechiae and encroach on the glomerular-tubular junction, leading to a narrowing and obstruction of the urinary orifice. These processes end in a decrease in filtrate delivery, finally depriving the tubule from any workload, causing atrophy and decomposition of the corresponding tubule. The affected kidney tissue undergo a series of events in attempt to repair from the damage, leading to production of proinflammatory cytokines, monocytes/macrophages, T cells, and stimulating mesangial cells, fibroblasts, and tubular epithelial cells to undergo phenotypic transition [4]. These processes lead to activation of matrix-producing effector cells and interstitial fibroblasts that induce the production of myofibroblasts, and later tubular epithelial to mesenchymal transition (EMT), with de novo expression of α-smooth muscle actin and overproduction of interstitial matrix components such as type I and type III collagen and fibronectin [4,22,23]. Other mediators of renal fibrogenesis are clearly necessary to induce renal fibrosis that encompasses glomerulosclerosis and tubulointerstitial fibrosis, and they undoubtedly interact with kidney resident cells including the glomerular podocytes.

How does the loss of podocytes connect with glomerular disease progression culminating in renal tissue fibrosis? In the acute phase of glomerular injury, there is already supra-regulation of pro-fibrotic factors in the podocyte, such as transforming growth factor β (TGF-β), a member of the TGF-β/activin/inhibin/nodal subfamily and the bone morphogenetic protein/growth and differentiation subfamily. TGF-β is a central mediator of fibrosis, which induced extracellular matrix expansion (ECM), cell differentiation and proliferation, apoptosis and epithelial to mesenchymal transition (EMT) [24]. EMT is regulated by the TGF-β/Smad signalling pathway and leads to morphological and phenotypic changes of the tubular epithelial cells, detachment from the tubular basement membrane and migration into the interstitium, where interstitial cells are transformed into myofibroblasts, inducing the synthesis and deposition of collagens and fibronectin [13,25]. Activation of TGF-β/Smad pathway signalling also induces supra-regulation of connective tissue growth factor (CTGF) and of vascular endothelium growth factor (VEGF), and both act by paracrine mechanisms on mesangial cells, stimulating the synthesis of ECM proteins and, later, inducing glomerulosclerosis and progressive renal fibrosis [26]. In addition to the canonical TGF-β/Smad pathway, TGF-β directly activates non-Smad signalling pathways, including the mitogen-activated protein kinase (MAPK), extracellular signal-regulated kinase (ERK) and phosphoinositide 3-kinase (PI3K) pathways, which have important roles in mediating podocyte injury and apoptosis, both in vitro and in vivo [27,28].

There is extensive evidence pointing to the involvement of TGF-β, CTGF, and VEGF in connection with glomerular podocytes in the pathogenesis of renal fibrosis, in both animal models and in humans with kidney diseases. In our study, patients with diabetic renal disease and those with podocytopathies, specifically FSGS, MN, and MCD, showed a significant upregulation of profibrotic inducers TGF-β, CTGF, and VEGF relative to control biopsies, and, at the same time, downregulation of mRNA of alpha-actinin-4, podocin, and podocalyxin. These findings are in line with those shown in other studies dealing with active and chronic glomerular diseases that progress to chronic damage and renal fibrosis. Experimentally, in acute and chronic puromycin aminonucleoside nephrosis as an animal model of MCD and FSGS, podocyte mRNA for CTGF, TGF-β2, fibronectin, and collagens I, III, and IV were overexpressed [29]. Once the chronic insult persists, there is a loss of actin fibres, accumulation of EMC, cell death, and progressive glomerulosclerosis. Clinical studies also reported the link between overexpression of profibrotic factors, podocyte damage, and glomerular and tubulointerstitial fibrosis in FSGS [30], MN [31], diabetic nephropathy [32], mesangioproliferative glomerulonephritis [33], and IgAN [34].

CTGF is a major autocrine growth factor induced by TGF-ß in podocytes. The expression of CTGF mRNA and/or protein in the mesangium and podocytes is upregulated in human chronic glomerular disease [14]. CTGF is a matricellular protein of the CTGF, Cyr61 and NOV (CNN) protein family that modulates and regulates the signalling of other growth factors, mediates ECM-cell communication and is identified as an important profibrotic factor in renal fibrogenesis [35]. CTGF mRNA and protein expression are restricted to podocytes in normal adult glomeruli. In rats treated chronically with puromycin, the CTGF mRNA was found to be highly upregulated in podocytes and correlated with proteinuria, a high expression of glomerular fibronectin, and collagens I, III, and IV, promoting progressive accumulation of the glomerular extracellular matrix in the mesangial area [29]. In another experimental model, Lu et al [15] showed that in spontaneously hypertensive rats, unlike the controls, CTGF, collagen III, and α-SMA were highly expressed in glomerular and tubular epithelial cells and in interstitial areas, with excess extracellular matrix deposition and glomerulosclerosis. The authors suggest that a disrupted interaction between podocytes and CTGF produces collagen deposition and transformation of epithelial and interstitial cells into myofibroblasts, thus causing interstitial fibrosis and glomerulosclerosis.

In our study, however, CTGF mRNA was the most weakly correlated of the mediators of kidney fibrosis we have measured. Thus, based in our findings, we cannot state that CTGF upregulation correlated with the extent of fibrosis as Sirius Red and HHF35 did. However, what does CTGF stain? In an experimental model in Sprague-Dawley rats, CTGF stained interstitial fibroblasts, coinciding with damage, regeneration, and fibrosis, as measured by smooth muscle actin, desmin, and vimentin immunohistochemistry [36]. In patients with ANCA-associated glomerulonephritis, CTGF immunostaining was strongly positive in podocytes and in cellular crescents, but not in fibrocellular or fibrous crescents, suggesting that CTGF could be a marker of fibrosis that is more active or young, and, therefore, may have greater potential for remodelling [37]. Ito et al. [38] measured CTFG mRNA expression by in situ hybridization in various glomerular diseases, showing a strong upregulation in the extracapillary and severe mesangial proliferative lesions of crescentic glomerulonephritis, LN, IgAN, FSGS, and diabetic nephropathy. These data suggest that CTGF is a marker of early stages of renal fibrosis, both in pro-inflammatory diseases and in non-proliferative, non-inflammatory glomerulopathies.

There are controversial data on the role of angiogenic factors, such as VEGF, in renal tubulointerstitial fibrosis. VEGF-A was the first described and best studied isoform, and its production by the podocyte is critical for endothelial cell migration, survival, proliferation, and differentiation within the glomerulus. In patients with diabetic nephropathy, a decrease in local VEGF-A production induces abnormal remodelling of the glomerular capillaries, endothelial cell loss, and progression to sclerosis [39]. In our study, VEGF-A was upregulated in DRD and in podocytopathies and correlated with HHF35, a mesenchymal marker that stains for α-smooth muscle actin. VEGF is a potent angiogenic molecule and is detected predominantly in podocytes, where TGF-ß1 stimulates its expression [40]. VEGF-A is probably activated more in early disease stages, where increased capillary density and vascular remodelling are most prominent, but its role in tubulointerstitial angiogenesis and fibrosis it is still unclear [41].

In this study, we reported an increase in mRNA expression of both CTGF and VEGF-A in biopsies of patients with DRD and podocytopathies, but not in patients with proliferative nephritis or IgAN. Different from our findings, Baelde et al. [32] reported a decrease in the mRNA levels of VEGF-A and CTGF, both in glomerulus and in whole renal cortex, in renal biopsies of patients with diabetic nephropathy compared to control kidneys. These authors described a positive and significant correlation between the reduced expression of VEGF-A and CTGF mRNA levels and of the podocyte proteins nephrin, podocin, and WT1, and an inverse correlation between podocin and the extent of interstitial fibrosis, as measured by Sirius Red and CD31 staining. In our study, we also found a reduced expression of podocyte proteins and an inverse correlation with the percentage of renal fibrosis that was significant for CTGF. Baelde et al. [32] suggested that VEGF-A and CTGF downregulation was connected to the podocyte loss occurring in more advanced stages of diabetic nephropathy. We argue that, in our patients with MCD, FSGS, and MN who presented an increased expression of profibrotic factors, the indication for a kidney biopsy was observed at an earlier stage of the disease. This is not the case for patients with DRD, as 69% were classified as Tervaert IIb, III or IV indicating more advanced stages of diabetic nephropathy, that is, severe mesangial expansion and nodular sclerosis [20].

The expression of TGF-β, CTGF and VEGF-A in kidney biopsies showed a positive and significant correlation with the measured percentage of renal fibrosis, being higher for HHF35, intermediate for Sirius Red, and lower for CTGF. HHF35 is a mesenchymal marker, as it stains for α-smooth muscle actin mainly in the early phase of fibrosis developing in multiple organs [19]. There should be a connection of α-smooth muscle actin linked to EMT. EMT transition causes loss of the epithelial cell markers podocin, nephrin, and E-cadherin, among others, and a gain of the mesenchymal markers α-smooth muscle actin, fibronectin, myofibroblasts, and type I collagen. Intrinsic fibroblasts can also alter their phenotype into myofibroblasts and, thereby, contribute to the fibrotic process. In the middle stages, TGF-β, matrix metalloproteinases, vasoactive molecules, and reactive oxygen species act as inducers of EMT, and this progressive process ends in tubulointerstitial fibrosis and glomerulosclerosis [41,42].

In our sample, Sirius Red showed the strongest and most extensive area of fibrosis under unpolarized light in all glomerular diseases. Compared to CTGF and HHF35, Sirius Red stained a higher percentage of glomerular and tubulointerstitial fibrosis, mainly in primary podocytopathies and in DRD. Interestingly, we found a negative correlation between positive area of fibrosis measured by Sirius Red with eGFR at the time of biopsy and at last follow up. Using eGFR as an independent indicator of kidney function, these findings suggest that the extent of kidney fibrosis may be a potential marker of deterioration of renal function, and progression to more advanced stages of chronic kidney disease [6].

Sirius Red has a high specificity for binding to collagen I and III fibres. Compared to CTGF and HHF35, Sirius Red stained a higher percentage of glomerular and tubulointerstitial fibrosis, both in primary podocytopathies and in DRD. Farris et al. [16] compared four morphometric techniques to measure renal fibrosis, including collagen III immunohistochemistry, trichrome and periodic acid-Schiff subtraction morphometry, and Sirius Red under unpolarized and polarized light in patients with primary and secondary glomerulopathies. Sirius Red stained fibrils in the interstitium, areas of intimal fibrosis, and fibrosis in the glomeruli and in arteries. Visual and morphometric techniques had good to excellent interassay and interobserver reproducibility and collagen III and unpolarized Sirius Red had the strongest correlations and the greatest dynamic range [16]. However, Sirius Red is not widely used as a standard method to measure renal fibrosis because it is more time consuming and expensive to both perform and to analyse [17]. Thus, a variety of techniques have been used to measure renal fibrosis, but no single technique can be described as the gold standard. In clinical practice, assessment of fibrosis using Masson trichrome staining is still the standard procedure, but it has been reported to be poorly reproducible among nephropathologists [16–18].

Inhibition of the TGF-β/Smad and CTGF pathways may be potential targets in the treatment of glomerular diseases, as suggested by initial clinical studies. The blockade of the TGF-β promoter by pirfenidone, a small synthetic molecule, in patients with FSGS decreased GFR loss by 25% [43]. Furthermore, the use of Fresolimumab, a high-affinity neutralizing antibody that targets TGF-β isoforms, has also been explored in patients with FSGS [44] but had no benefit on partial or complete disease remission, though a trend to stabilized eGFR in the Fresolimumab group was described. In published experimental studies, it has been demonstrated that the suppression of the TGF-β1/Smad3 [45] pathway significantly attenuates renal fibrosis by the ablation of profibrotic regulators. Treatment with the CTGF antisense oligonucleotide reduced expression of genes involved in matrix expansion (fibronectin, collagen I and IV), and of an inhibitor of matrix degradation in the renal cortex, thus contributing to a significant reversal of mesangial expansion in two experimental models of diabetic nephropathy [46]. In a clinical study, the inhibition of CTGF using FG-3019 (a human monoclonal antibody) in patients with diabetic kidney disease decreased albuminuria [47].

This study has some limitations. Overall, seventy-two percent of the biopsies in our study where done less than one year from clinical disease manifestations, that is, in more early stages of glomerular and tubulointerstitial damage. If we had included a greater proportion of biopsies done after three or even five years of the initial disease manifestations, we could capture more advanced stages of glomerulosclerosis and tubulointerstitial fibrosis, with a higher index of kidney fibrotic damage and potentially a better correlation with the progression of chronic kidney disease. In addition, most of the patients were under immunosuppressive treatment that certainly can modify the molecular mechanisms inducing renal fibrosis. The predominant histological lesions of our sample expressed more an acute reaction of the glomerulus to injury, such as mesangial matrix expansion, mesangial hypercellularity, thickening of MBG, and inflammatory cellular infiltration, than chronic damage. Indeed, in 58% of the biopsies, global glomerulosclerosis was present in less than 30% of the glomeruli. Interstitial fibrosis and tubular atrophy, as semi-quantified by the nephropathologist, were mild in 57% of the patients (5–25%), and IFTA was moderate (26–50%) or severe (>50%) in only 37% of patients. Interestingly, in biopsies diagnosed as MCD, FSGS, MN, and DRD, mRNA of alpha-actinin-4, podocin, and podocalyxin were downregulated and, at the same time, the mRNA of TGF-β1, CTGF, and VEGF-A were upregulated, suggesting that, after acute podocyte injury, the inductors of tissue fibrosis were highly activated.

## Conclusions

In these patients with glomerular diseases of different etiologies, the expression of profibrotic and angiogenic factors correlated positively with the percentage of renal fibrosis as quantified by HHF35, Sirius Red, and CTGF staining. Despite significant advances in the understanding of the mechanisms and molecular pathways of renal fibrosis, the translation to clinical trials is still scarce. As recently reviewed [48,49], the use of effective antifibrotic treatments to slow progression or even reverse the chronic kidney injury in glomerular diseases is a great challenge to be explored in future clinical trials.
